# Supplementary figures and images for: Epitope-targeting platform for broadly protective influenza vaccines
Source: PLoS One. 2021 May 27;16(5):e0252170. doi: 10.1371/journal.pone.0252170 (PMC8158873; doi:10.1371/journal.pone.0252170)

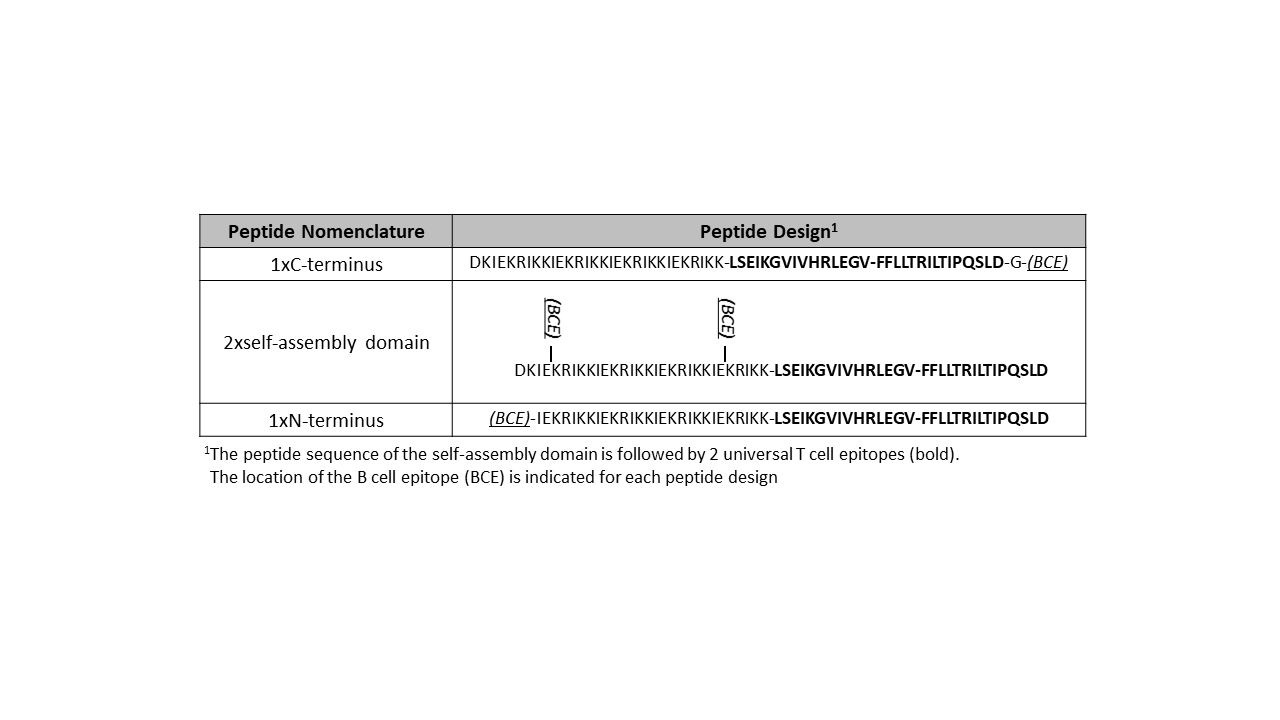

Supplement: S1 Table — (TIF) [file pone.0252170.s001.tif]

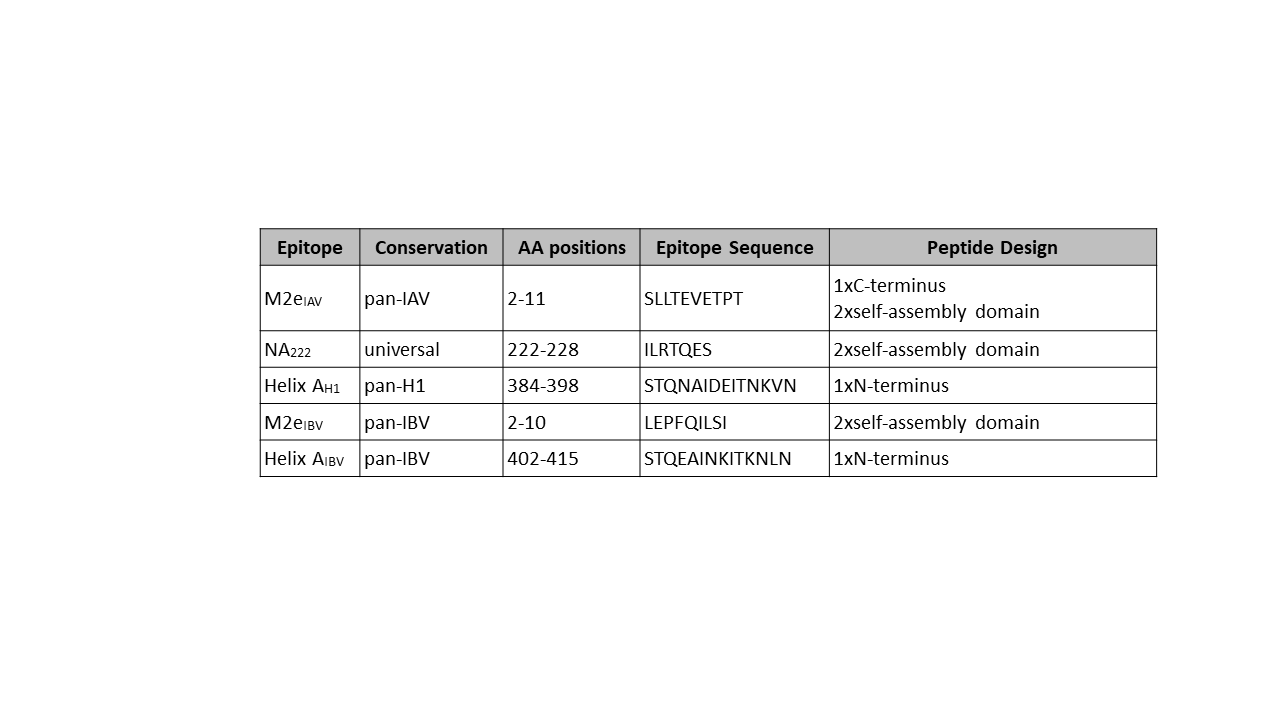

Supplement: S2 Table — (TIF) [file pone.0252170.s002.tif]

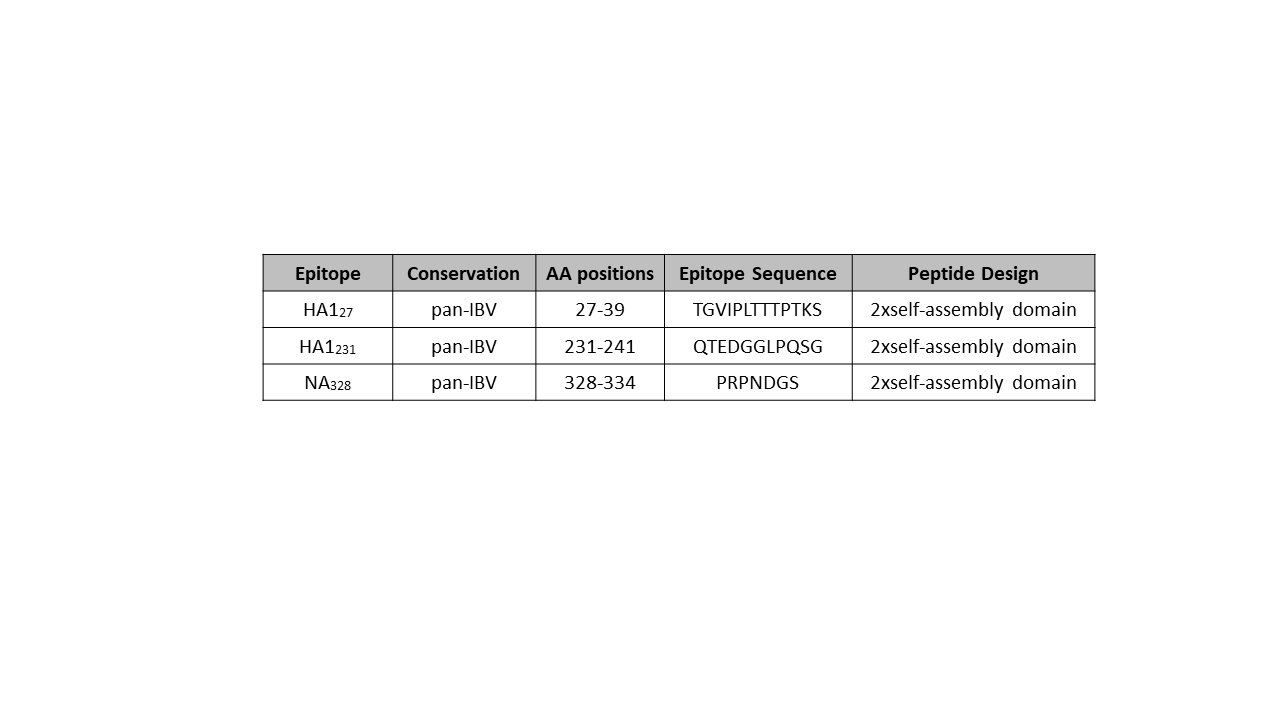

Supplement: S3 Table — (TIF) [file pone.0252170.s003.tif]

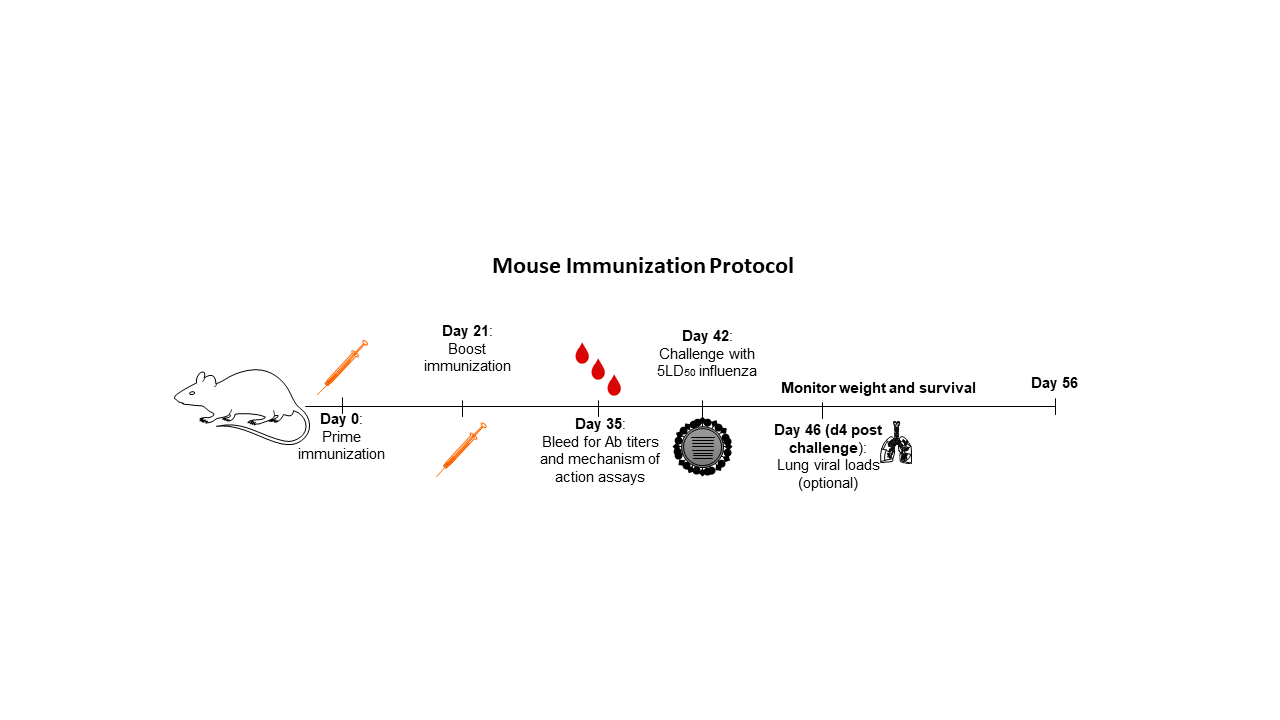

Supplement: S1 Fig — (TIF) [file pone.0252170.s004.tif]

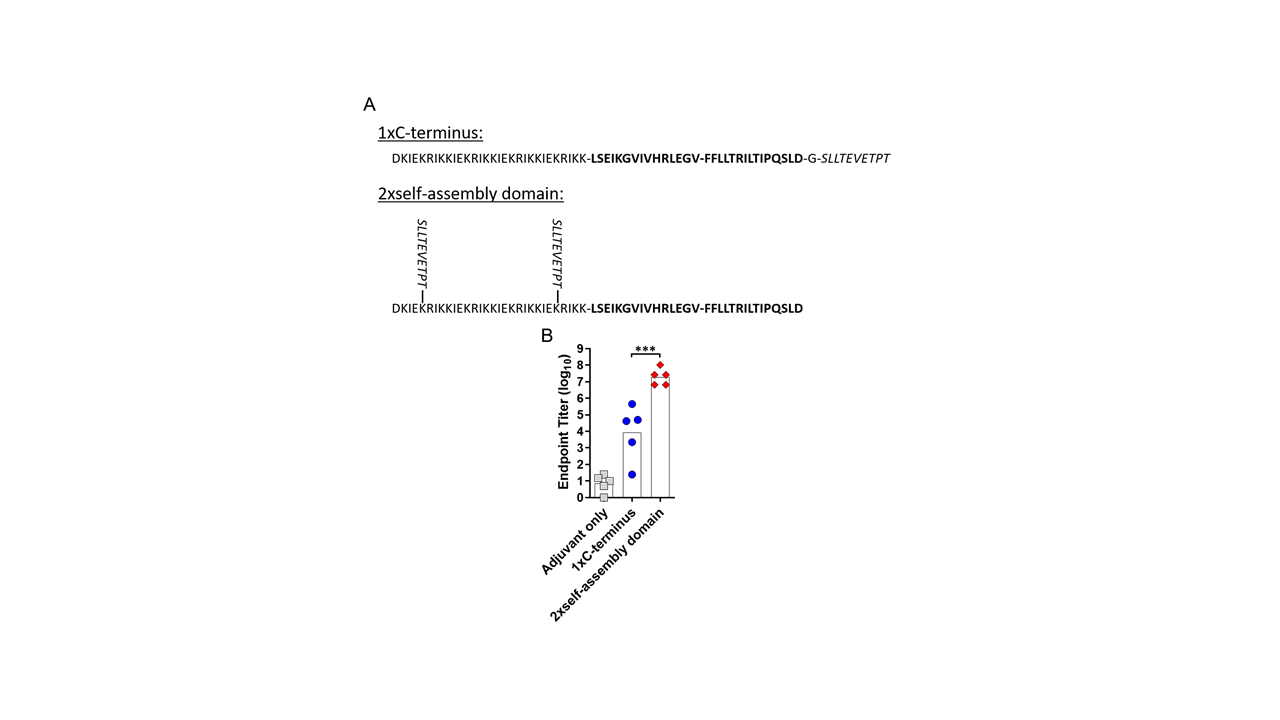

Supplement: S2 Fig — (A) Amino acid sequences of M2e antigens. The M2eIAV epitope (italics) was synthesized onto the C-terminus of the peptide monomer (1xC-terminus) or grafted onto two lysine sidechains within the self-assembly domain using isopeptide bonds (2xself-assembly domain). CD4 T cell epitopes from Measles and Hepatitis B are shown in bold. (B) Immunogenicity of peptides. CD-1 mice (n = 5) received a prime-boost immunization with GLA-SE (or GLA-SE only) and d35 titers were assayed by ELISA. A one-way analysis of variance (ANOVA) followed by Tukey’s multiple comparisons test was used for statistical analysis (***P<0.001). (TIF) [file pone.0252170.s005.tif]

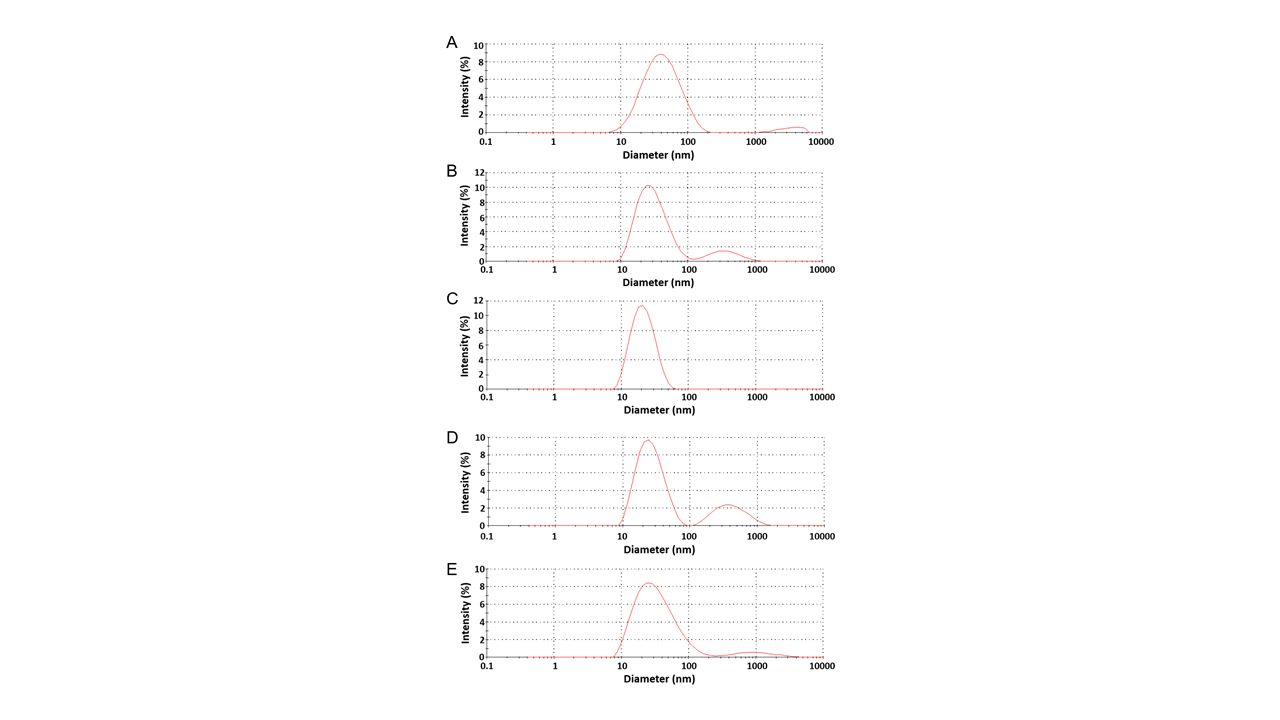

Supplement: S3 Fig — Dynamic light scattering was used to verify nanoparticle size of (A) M2eIAV, (B) NA222, (C) Helix AH1, (D) M2eIBV, and (E) Helix AIBV. (TIF) [file pone.0252170.s006.tif]

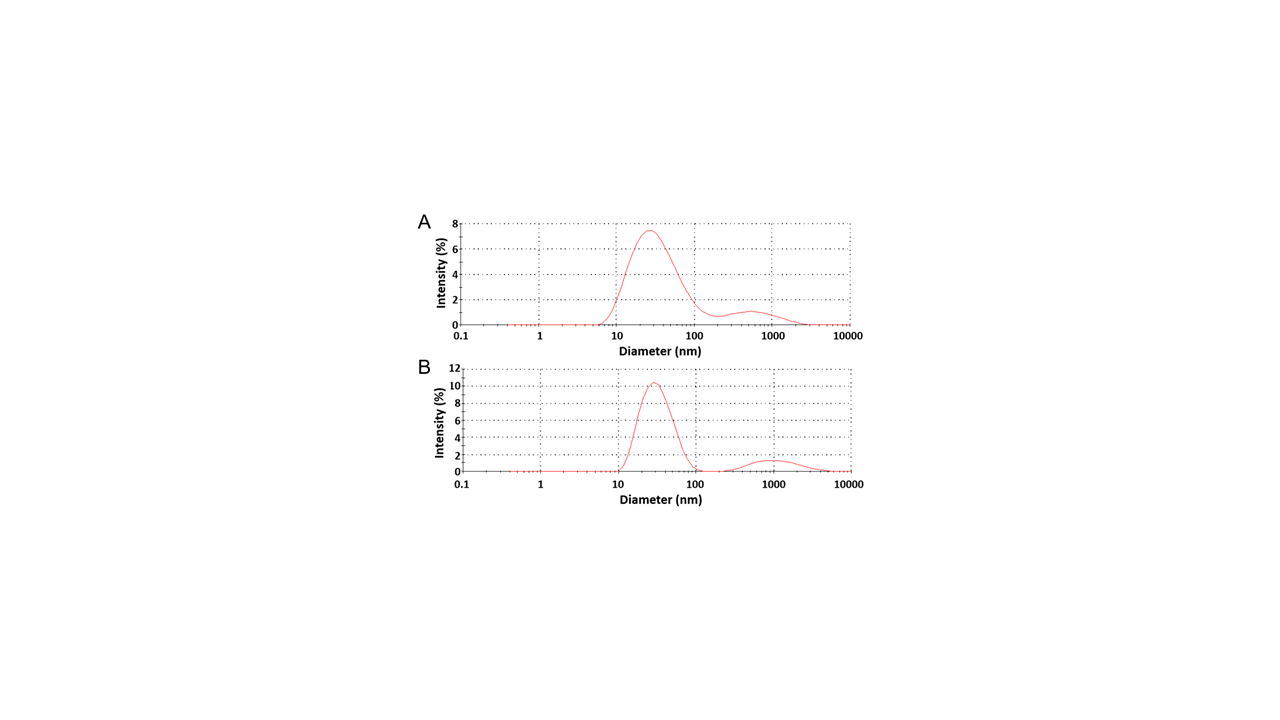

Supplement: S4 Fig — Dynamic light scattering was used to verify nanoparticle size of (A) M2eIAV + Helix AH1 and (B) M2eIBV + Helix AIBV formulations. (TIF) [file pone.0252170.s007.tif]

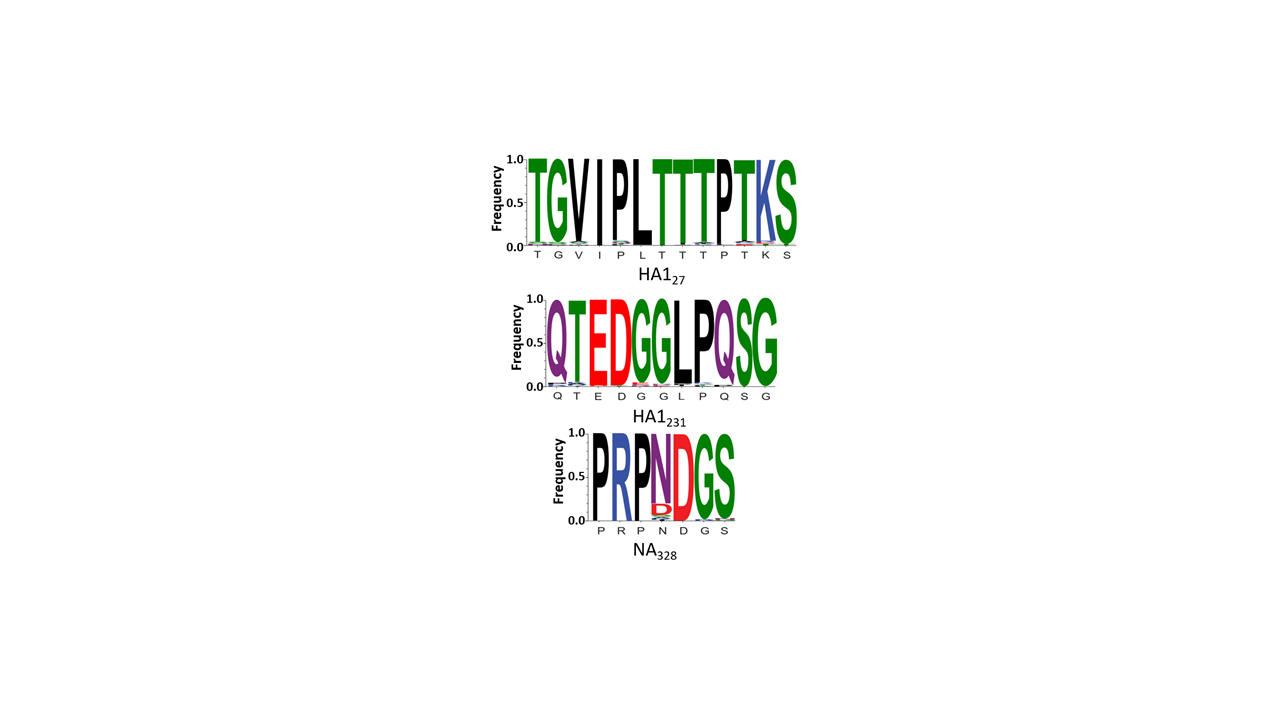

Supplement: S5 Fig — The amino acid sequence of each epitope is depicted, with the residue letter height proportional to its mutational frequency in aligned HA or NA sequences. Amino acids are colored according to chemical properties: green (hydrophilic), black (hydrophobic), red (acidic), and blue (basic). (TIF) [file pone.0252170.s008.tif]

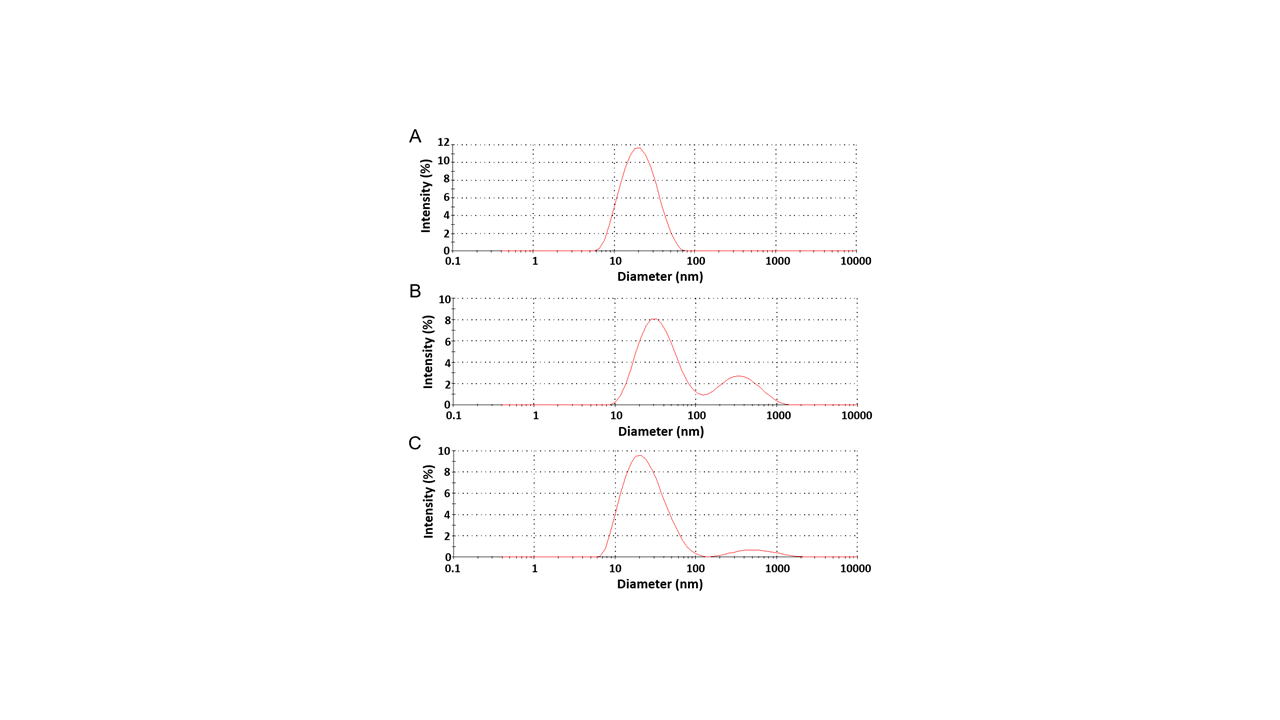

Supplement: S6 Fig — Dynamic light scattering was used to verify nanoparticle size of (A) HA127, (B) HA1231, and (C) NA328. (TIF) [file pone.0252170.s009.tif]

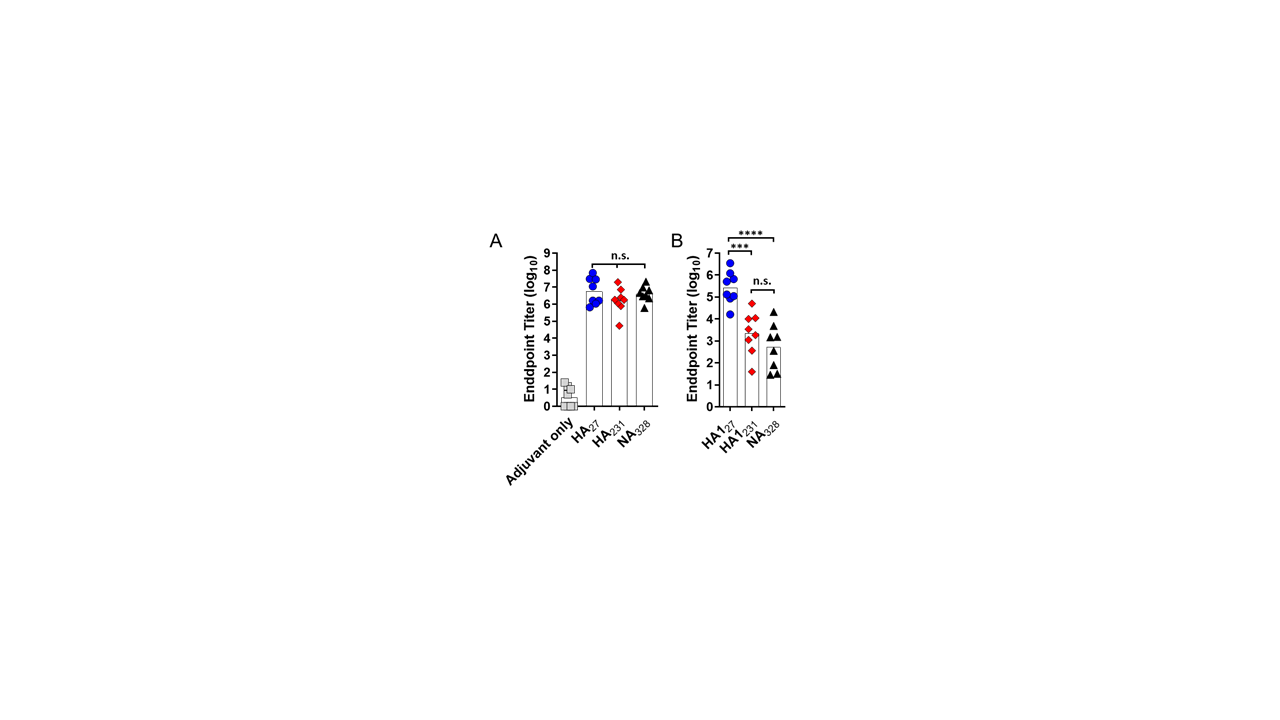

Supplement: S7 Fig — CD-1 mice (n = 8) were immunized the indicated peptide plus GLA-SE (or GLA-SE only). Antisera (d35) from each group was screened for titers to (A) BSA-epitope conjugates or (B) recombinant HA/NA. One-way ANOVA followed by Tukey’s multiple comparisons test was used for statistical analysis of titers (***P<0.001, ****P<0.0001, n.s. not significant). (TIF) [file pone.0252170.s010.tif]

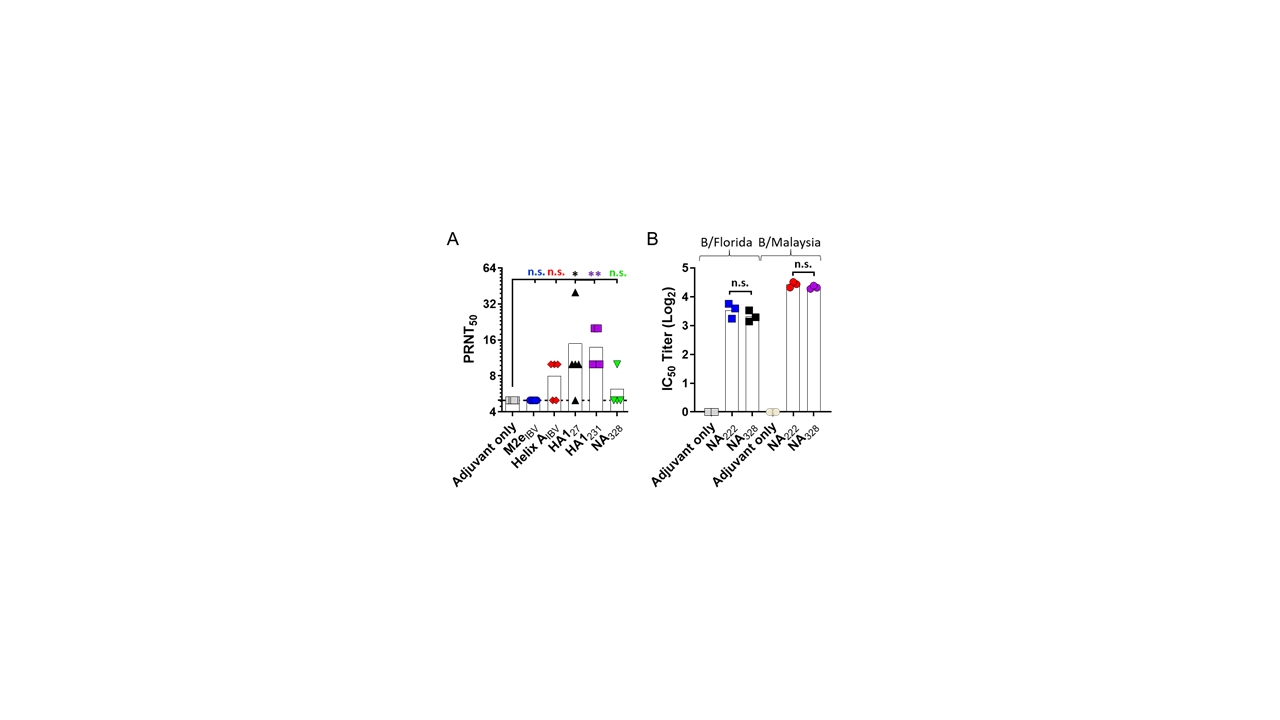

Supplement: S8 Fig — (A) Plaque reduction neutralization titers. CD-1 mice (n = 5) were immunized (d0, d21) with the indicated peptide plus GLA-SE (or GLA-SE only). Day 35 antisera was assayed for neutralizing activity in a PRNT assay. One-way ANOVA followed by Dunnett’s multiple comparisons test was used for statistical analysis between control and indicated test group (*P = 0.0156, **P = 0.0044, n.s. not significant). Limit of detection depicted with dashed line. (B) NA neutralizing ability. CD-1 mice (n = 3) were immunized as above. Day 35 antisera was assayed for its ability to prevent cleavage of an NA substrate (see Materials and methods). One-way ANOVA followed by Tukey’s multiple comparisons test was used for statistical analysis. Color coded asterisks without brackets denote significance between control and indicated test group; brackets indicate comparison between test groups (*P<0.05, **P<0.01, n.s. not significant). (TIF) [file pone.0252170.s011.tif]

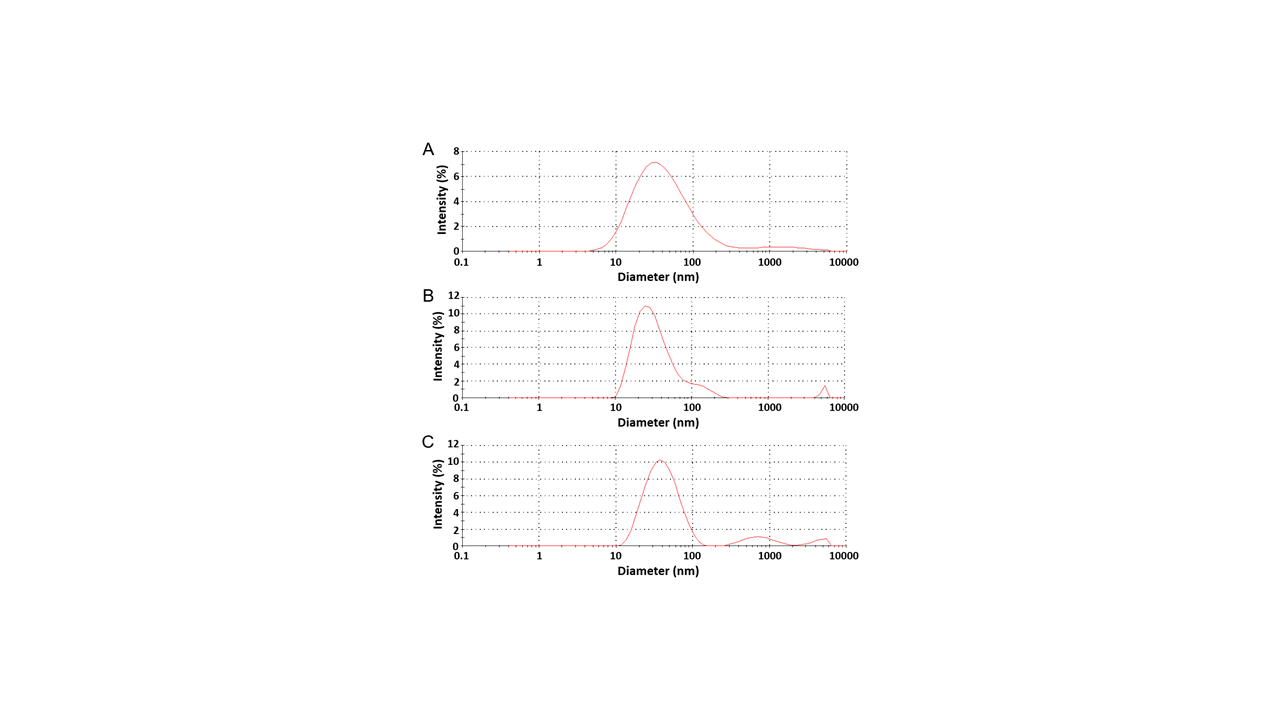

Supplement: S9 Fig — Dynamic light scattering was used to verify nanoparticle size of (A) HA127 + M2eIAV, (B) HA127 + Helix AH1, and (C) HA127 + HA1231 formulations. (TIF) [file pone.0252170.s012.tif]
