# Supplementary material for: Epitope-targeting platform for broadly protective influenza vaccines
Source: PLoS One. 2021 May 27;16(5):e0252170. doi: 10.1371/journal.pone.0252170 (PMC8158873; doi:10.1371/journal.pone.0252170)
Supplement: Attachment — Submitted filename: Response to Reviewers.docx [file pone.0252170.s013.docx]

December 9, 2020

Dear Dr. Liu,

We thank the reviewers for their insightful comments and have revised the manuscript to improve its suitability for publication in *PLOS Pathogens*.

***Responses to the reviewer’s major comments include the following:***

*Reviewer #1:*

*1. Findings of this study using HA2 and/or M2e peptides to protect against homologous and heterologous influenza viruses are not new and have been previously described. Key previous work was not properly cited (e.g. PMID: 20956293; PMID: 18343837; PMID: 25799221).*

Response: The Reference section has been extensively revised to include these citations and additional relevant work.

*2. The authors claim several times “broadly protective influenza vaccines.” However, they tested only one influenza A and two influenza B. What about H3N2 virus or other zoonotic viruses (e.g. swH1N1, H5N1, H7N7, H9N2, etc.)? Protection against other strains should be also tested.*

Response: We appreciate this perspective and have removed this phrase from the Abstract and Introduction to prevent any misunderstanding. The presumption of broad protection is based on our technology’s ability to target highly conserved epitopes. We first tested the relative activity of known IAV and IBV epitopes and then used this information to discover and down select a new Influenza B vaccine candidate. The M2e, NA, and Helix A sequences are highly conserved across subtypes and their ability to induce broadly protective antibodies is well grounded in the literature. By extension, the novel influenza B sites are >99% identical across IBV strains, which strongly suggests that protection against one strain should translate to others, barring major structural differences in the sites. Given the wide scope of the suggested studies, the number of antigens involved and associated cost, we felt the complexity of running parallel heterologous challenges was better suited to another manuscript with a narrower focus on more finalized vaccine candidates.

*3. Lines 160, 213-219, 230 and 262: These hypotheses are not enough. The mechanism of action of these peptides should be studied in more details. Further experimentations are needed to understand the mechanism for protection: how does the anti-M2e antibody work (e.g. virus entry, release, interaction with the immune system), likewise, how does the anti-HA127 antibody work (e.g. prevent cleavage-activation, fusion activity, budding)?*

Response: The findings being reported have generated many new questions and we agree that the mechanism of antibody protection should be further investigated for previously unknown epitopes, especially HA1_27_. However, given the breadth of our current data set, we focused on a preliminary classification of antisera into directly HA/NA neutralizing or non-neutralizing. We respectively submit that the mechanistic studies as described by the reviewer would be better suited for a subsequent more in-depth publication. We address the importance of these studies in the Discussion section.

*4. Studies using different mutations in these peptides (Aka antigenic drift) should be studied.*

Response: The reviewer raises an important avenue of research. One question addresses each epitope’s mutation susceptibility, and the other, the degree of sequence mismatch that is tolerable between the vaccine antigen and mutated epitope for effective antibody binding. These are testable questions, but they will require a careful and more thorough analysis, which we submit, goes beyond the scope of our present work. We address the importance of these studies in the Discussion section.

*5. The results section is narrative and it was very difficult to extract the results. Likewise, The discussion section is also narrative and should be shortened.*

Response: We agree that this was a problem. The Results section is now streamlined, and the Discussion completely re-written to simplify experimental rationale and interpretation.

*Reviewer #3:*

*1. ADCC experiment should be conducted for those non-neutralizing or non-neuraminidase inhibiting groups.*

Response: We agree that a more thorough evaluation of antibody MOA is important, but we respectively submit that this information is not required for interpreting the performance of this novel technology. ADCC-based protection of M2e is clearly established in the literature and our data indirectly verified this since the M2e antisera showed no PRNT signal. Our preference is to characterize the antibodies targeting all of these epitopes more carefully using a variety of assays in a subsequent set of experiments.

*2. Only one virus for each subtype was used in challenge studies, which is not sufficient to draw a conclusion for "universal" protection.*

Response: We agree that our use of “universal” was misleading and have removed it from the text.

*3. TEM imaging should be done to justify the described peptide nanoparticles.*

Response: We reference our previous publication (Zeigler et al. npj Vaccines 2019) where nanoparticle sizes were confirmed using orthogonal techniques; dynamic light scattering, TEM, and analytical ultracentrifugation.

Responses to the Reviewers minor comments include the following:

Reviewer #1:

*Line 50 and 52: please cite more suitable references for these sentences.*

Response: The updated reference section corrects this oversite.

*Line 224: These data should be shown, at least as supplementary.*

Response: This was an error and the sentence has been deleted.

*Line 329: Animals, please write the number of animals used in each experiment. It is not clear if the lungs were taken from one or more than one of the combined experiments.*

Response: This information has been included in the revised methods section

*Line 236: Are amino acids of these HA1 peptides involved in antigenic epitopes (e.g. epitopes A-E)? The antigenic drift can happen due to direct mutations not only through (de)glycosylation.*

Response: Based on Sun et al. J Virol 93:e01673-18, 2018, the HA1 epitopes are outside of the major antigenic sites on HA. This reference has been added to the manuscript.

Reviewer #2:

*The authors should consider inserting subheadings in the Results section for greater clarity.*

Response: Subheadings are now included in the revised Results section.

Reviewer #3:

1. Why were CD-1 mice used rather than BALB/c mice, which are more common in flu virus study?

Response: Our peptide vaccines use CD4 T cell epitopes that bind promiscuously to a broad repertoire of MHCII molecules. Outbred CD-1 mice are used, in contrast to BALB/c, to more accurately model the genetic diversity of the human population. This rationale has been included in the methods section. Our limited in-house studies using C57BL/6 mice generated similar results.

2. Can authors clarify the specificity of CD4 T cell epitopes used in this study for CD-1 mice ? Please also discuss the translational aspects of these CD4 T cell epitopes for human use.

Response: This manuscript refers to our previous publications (Miller et al 2014, Zeigler et al, 2019) that report the selection and activity of these epitopes in multiple strains of mice and in rats. Regarding translatability, we have recently completed a nicotine vaccine experiment in cynomolgus macaques that tested three different sets of T cell epitopes. Importantly, most of the animals, including those that used the epitopes described in this manuscript, induced substantial antibody concentrations (>10 ug/ml sera). This result bodes well for future clinical studies and is the subject of a manuscript in preparation.

3. Authors should move some of the titering data to the main figures.

Response: These data are now shown in new Figures 1 and 3.

4. Did authors examine the antibody binding specificity against the whole virions ? or authors may want to discuss possible differences when using peptides/recombinant proteins/whole virions as coating antigens in ELISA.

Response: We have tested ELISAs with whole virions but these data were not discussed since they didn’t provide any more information than recombinant/conjugate detection reagents. The Discussion section contrasts recombinant/conjugate ELISA responses.

5. Line 113-->Fig. 3A.

Response: Thank you, this mistake has been corrected.

6. For the DLS results, there are several unusual peaks (aggregations) beyond the 100 nm, such as the Fig. S2D and S7B. Authors please justify the formations of nanoparticles.

Response: The weighting toward larger species in DLS intensity spectra suggests these aggregates represent a very small proportion of assemblies. This was corroborated by analytical ultracentrifugation (Zeigler et al. npj Vaccines 2019). Future studies will determine whether these minor species are byproducts from the solid phase synthesis reaction and/or the formulation conditions.
