# Supplementary material for: Epitope-targeting platform for broadly protective influenza vaccines
Source: PLoS One. 2021 May 27;16(5):e0252170. doi: 10.1371/journal.pone.0252170 (PMC8158873; doi:10.1371/journal.pone.0252170)
Supplement: Attachment — Submitted filename: Response to Reviewers_.docx [file pone.0252170.s014.docx]

April 6, 2021

Dear Dr. Huber,

We thank the reviewers for their insightful comments and have revised the manuscript to improve its suitability for publication in *PLOS ONE*. We would certainly like to be included in the upcoming Influenza Collection. The manuscript has been updated according to the established guidelines, including formatting, peptide sequences/designs, and mouse euthanasia procedures. As requested, we have revised the funding and competing interests statement as follows:

Funding Statement:

This research was supported in its entirety by the Center of Disease Control ([www.cdc.gov](http://www.cdc.gov)) under award number R43IP001108 (CC). The funders had no role in the study design, data collection and analysis, decision to publish, or preparation of the manuscript. The authors are employed by TRIA Bioscience Corp, which provided support in the form of salaries but did not have any additional role in the study design, data collection and analysis, decision to publish, or preparation of the manuscript. The specific roles of these authors are articulated in the ‘author contributions’ section. There was no additional external funding received for this study.

Competing Interests:

All authors are employees of TRIA Bioscience Corp. DZ and CC are co-inventors on a pending patent (Applicant: TRIA Bioscience Corp., “Synthetic carrier compositions for peptide vaccines”, WO/2020/047107) pertaining to peptide composition of matter and methods of use. This does not alter our adherence to PLOS ONE policies on sharing data and materials.

***Responses to the reviewer’s comments include the following:***

**Reviewer #1:**

1. *Lines 113-114 the sentence is incomplete.*

Response: This sentence has been modified.

*2.	Line 148. Please indicate the sex and age of CD-1 mice used in the study. In addition, it would be beneficial for the paper if the authors added a figure with mouse study design, so that the procedures were easier to follow.*

Response: Information about the CD-1 mice is updated in the Materials/Methods section and a new supplemental figure (S1 Fig.) describes the mouse study design.

3. *Lines 151-155. There should be two separate sentences.*

Response: This error has been corrected.

*4.	Lanes 182-183. Please provide a reference for ELISA method or indicate how the endpoint titers were calculated. Were these antibodies of IgG isotype?*
Response: This information and a reference has been added to the Materials/Methods section.

*5.	Figures 1 and 3. Please specify that Y axes show IgG antibody titers.*

Response: The Y axes have been corrected in new fig 1, 2, and 4.

*6.	References 24, 44 are incomplete. Please correct.*

Response: These references have been updated.

**Reviewer #2:**

*Many figures are small, with a single panel or just a few panels, with additional figures in the supplement. I would suggest grouping into multipanel figures and moving as much SI data to the main text as allowable, to make it easier for the reader. For example, Figs 1+2 could be grouped, Figs 3+4 (or even Figs 1+2+3+4); Figs 5+6+S7. Some things, like assessing nanoparticle size/integrity makes sense in the SI,*
Response: As suggested, Figs 1+2 are now grouped, as well as Figs 3+4, Figs 5+6, and Figs 7+8+S9 Fig.

*Lines 29-30: In silico method: I think this statement should be removed from the abstract. It makes it sound like a new computational tool was developed for epitope selection, but ultimately seemed to be making educated guesses based upon structural information, which is not new.*

Response: This statement has been modified to make it clear that this is not a new strategy.

*Lines 269-272: “This clearly demonstrates the advantage of targeting influenza epitopes simultaneously.” Fig. 4 shows that the bivalent formulation protects mice better than adjuvant only, but it doesn’t show a comparison between the bivalent and monovalent formulations or whether the bi/monovalent difference is statistically significant. Authors should revise this claim or provide evidence.*

Response: This sentence has been revised as suggested.

*Lines 356-358: “This evidence confirms…” I would suggest softening this statement. In the same figure there are other examples where targeting 2 epitopes did not result in significantly reduced titers relative to HA1(27) alone, and the HA1(27) + Helix AIBV combination is only significant at the p<0.05 level. So while I agree with the authors overall interpretation of their data, I would say it isn’t clear how generally true this will be with bivalent formulations.*

Response: This assertion has been softened.

**Reviewer #3:**

1. *It is difficult to follow this manuscript because of insufficient information on the peptide vaccines tested in this study. The S1 table should be extended to include more details in all peptide vaccines tested: amino acid position (either HA Head or stem) and then consistent numbers of start and end in the peptide epitope sequence given (Helix A-H1, Helix A-IBV, etc.)? – Fig 5 or Fig 6 needs to provide information on the flu B peptide sequences for HA1-27, HA1-231, NA328.*

Response: Thank you for this feedback. We now include a new S1 Table depicting the general peptide designs and location of the B cell epitopes (BCE), a new S2 Table that more fully describes the previously known influenza epitopes, and a new S3 Table that contains information about the new IBV epitopes.

2. *It is unclear how much is the sequence homology between the conserved peptide vaccines and challenge viruses. Are all peptide sequences 100% conserved in the challenge viruses tested?*

Response: This has been clarified in the text.

3. *Fig 6 data on lung viral titers in TCID50 after challenge with influenza B/Florida: HA1-27 +Helix A-IBV, HA1-27 +M2e-IBV, HA1-27 +HA1-231 show corelates with weight changes (Fig 7). However, the M2e-IBV+ Helix A-IBV (~2 or 3% weight loss, Fig 4) and HA1-27 (7 or 8% weight loss) are not showing correlates with those in groups shown in the Fig 7. Please discuss this apparent discrepancy between body weight changes and lung viral titers.*

Response: This is a good observation. We ascribe this slight discrepancy to several things: 1) the use of outbred mice, which exhibit more variable responses to influenza challenge, 2) as mice succumb to the influenza challenge, their body weight is removed from the weight loss tabulation, which artificially decreases apparent weight loss in certain groups, 3) pulmonary viral titers are based on three randomly-chosen mice that are not included in the weight loss/survival plots -- thus, they are not fully representative of the rest of the cohort.

4. *The peptides vaccines contain a self-assembling domain (?) and non-flu pan-T cell epitopes, which is an approximately over 60 residues. Did the authors determine IgG antibodies recognizing the non-flu residues in the vaccines? Are the ELISA coating antigens the same as the vaccine peptides (with a self-assembling domain and non-flu pan-T cell epitopes) or peptide epitopes only specific?*

Response: This is an interesting question that is relevant to the translational potential of the technology. We have previously characterized and reported relative amounts of antibodies elicited to the self-assembly domain and TCEs of the Helix A antigen (Zeigler et al, 2019). Overall, the vaccines elicit >10-fold more antibodies to the BCE than the rest of the peptide. The exact difference varies by vaccine antigen. The ELISA reagents are described in the Materials/methods section.

*5.	The S1 Fig data showed that Increasing M2e epitope valency improved antibody responses. It is not clear whether M2e-IAB (or M2e-IBV, other peptides) contains one copy or 2 copies tested in all other figures data presented.*

Response: Please see new S2 and S3 Tables.

*6.	S3 Fig, S5 Fig, S8 Fig Nanoparticle Size distributions: Did the authors include soluble peptides samples (measure the sizes) as a negative control?*

Response: Although this was not a part of the current body of work, we have previously verified that soluble peptides do not exhibit Gaussian DLS distributions in this size range and have included this statement in the text.

*7.	S4 Fig provides informative Evolutionary sequence profiles of new IBV antibody targets. It will be informative if evolutionary sequence profiles are similarly presented for other peptides vaccines (Fig 1) used in this study.*

Response: The new IBV epitopes evolutionary profiles were shown to support our contention that they represent ideal targets for a “universal” IBV vaccine. Information about the sequence diversity of the other epitopes is the subject of numerous publications, some of which have been referenced in our work.

8.	*Vaccine doses: Does the bivalent peptide vaccine contain 5 ug each to make it 10 ug in total peptide vaccine dose?*

Response: The bivalent vaccines utilized 10 ug/peptide for a total of 20 ug. This information has been included in the Materials/Methods section. In-house studies comparing the 20 ug bivalent to 20 ug monovalent formulations have verified that the improvement in protection is not dose-related. This context has been included in the manuscript text.
